# Retraction: Diallyl Disulfide Suppresses SRC/Ras/ERK Signaling-Mediated Proliferation and Metastasis in Human Breast Cancer by Up-Regulating miR-34a

**DOI:** 10.1371/journal.pone.0295991

**Published:** 2023-12-12

**Authors:** The PLOS ONE Editors

After this article [1] was published, concerns were raised about some of the western blot panels in Fig 4D. Specifically:

- The β-actin vector SRC panel is rotated by 180° and duplicated as the β-actin scramble miR-34a panel.
- The BRCA1 panel in Fig 4A in [2] is adjusted, mirrored vertically, and duplicated as the Ras-GTP mock DADS panel in Fig 4D in [1].

In response to queries about the experiments in Fig 4D in [1], the corresponding author stated that the β-actin vector SRC panel was duplicated as the β-actin scramble miR-34a panel in error, and that the BRCA1 panel in Fig 4A in [2] was duplicated as the Ras-GTP mock DADS panel in Fig 4D in [1] in error. The corresponding author stated that the original data underlying the SRC panel in Fig 4C and the Ras-GTP vector SRC and ERK mock DADS panels in Fig 4D are no longer available. The corresponding author provided the original data underlying the remainder of Fig 4, but the *PLOS ONE* Editors have concerns about the authenticity of these images.

In addition, during editorial follow-up, similarities were noted between this article [1] and submissions and communications by other research groups.

In light of the above issues, which could not be resolved with the underlying data provided, and concerns around data handling, the *PLOS ONE* Editors retract this article.

HT, XXiao, and FY did not agree with the retraction. BC, XL, PL, GZ, and XXie either did not respond directly or could not be reached.

HT stands by the article’s findings.

## References

[1] Xiao X, Chen B, Liu X, Liu P, Zheng G, Ye F, et al. (2014) Diallyl Disulfide Suppresses SRC/Ras/ERK Signaling-Mediated Proliferation and Metastasis in Human Breast Cancer by Up-Regulating miR-34a. PLoS ONE 9(11): e112720. 10.1371/journal.pone.0112720 25396727 PMC4232521

[2] Wu Jiali, Shuang Zeyu, Zhao Jianfu, Tang Hailin, Liu Peng, Zhang Lijuan, Xie Xiaoming, Xiao Xiangsheng, Linc00152 promotes tumorigenesis by regulating DNMTs in triple-negative breast cancer, Biomedicine & Pharmacotherapy, Volume 97, 2018, Pages 1275–1281, ISSN 0753-3322, 10.1016/j.biopha.2017.11.055 29156515

